# Complete Genome Sequence of Flavobacterium sediminilitoris YSM-43^T^, Isolated from Tidal Sediment in Yeosu

**DOI:** 10.1128/mra.00054-22

**Published:** 2022-08-22

**Authors:** Pyeong An Lee, In-Tae Cha, Ki-Eun Lee, Youn Kyoung Son, Jaewoong Yu, Jin Nam Kim, Donghyeok Seol

**Affiliations:** a Center for Microbial Analysis, Korea Research Institute of Biomedical Science, Daejeon, Republic of Korea; b National Institute of Biological Resources, Incheon, Republic of Korea; c eGnome, Inc., Seoul, Republic of Korea; d Department of Agricultural Biotechnology, Seoul National University, Seoul, Republic of Korea; e Research Institute of Agriculture and Life Sciences, Seoul National University, Seoul, Republic of Korea; University of Delaware

## Abstract

Here, we report the complete genome sequence of Flavobacterium sediminilitoris YSM-43^T^, isolated from a tidal flat in Yeosu, Republic of Korea. The whole genome consists of one circular chromosome of 3,913,692 bp. A total of 3,599 genes were predicted, comprising 3,537 coding DNA sequences (CDSs), 50 tRNAs, 9 rRNAs, and 3 noncoding RNAs (ncRNAs).

## ANNOUNCEMENT

The genus *Flavobacterium*, the type genus of the family *Flavobacteriaceae* in the phylum *Bacteroidetes*, is a Gram-negative, yellow-pigmented, and rod-shaped bacterium. Commonly, *Flavobacterium* thrives in various habitats, including both terrestrial and marine ecosystems (1). At the time of writing, 268 valid descriptions of *Flavobacterium* species have been published (https://lpsn.dsmz.de/genus/flavobacterium) (2). According to BacDive (3), two *Flavobacterium* spp. have been isolated from tidal flats, Flavobacterium sediminis and Flavobacterium sediminilitoris (4, 5). *F. sediminilitoris* YSM-43^T^ was isolated from a tidal flat in the Republic of Korea in 2018, but its genomic properties are still unknown (5). Therefore, in this study, whole-genome sequencing of *F. sediminilitoris* YSM-43^T^ was conducted, which will provide insight into the adaptation of YSM-43^T^ to the tidal flats and promote future comparative genomic studies of bacteria in tidal flats.

**FIG 1:**
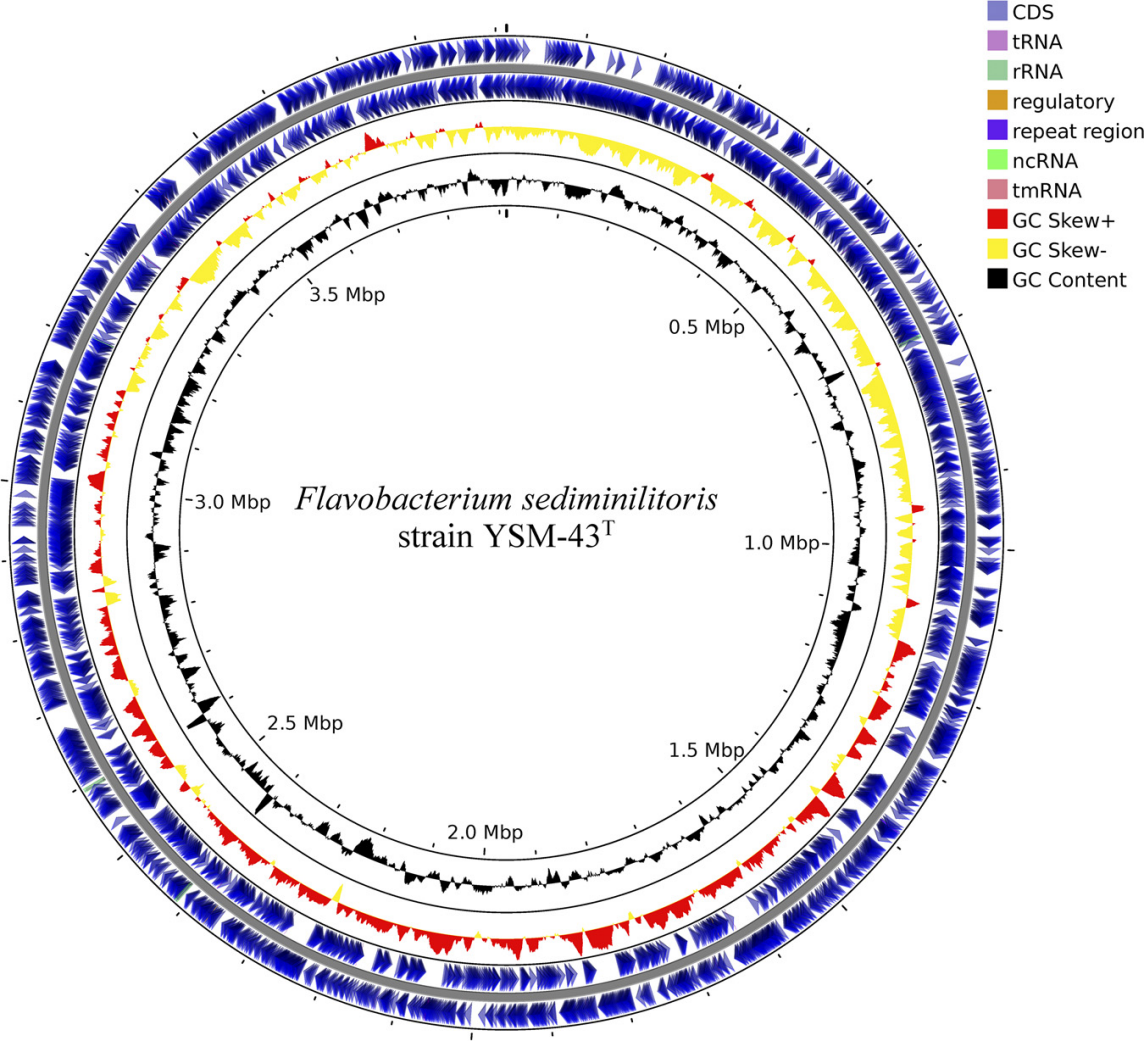
Circular genome map of the *Flavobacterium sediminilitoris* YSM-43^T^ chromosome: (outer to inner) CDSs on the forward strand, CDSs on the reverse strand, positive (red) and negative (yellow) GC skew, GC content (black). CDSs, coding DNA sequences; ncRNA, noncoding RNA; tmRNA, transfer-messenger RNA.

YSM-43^T^ was cultivated on marine agar 2216 (BD). The cells were collected in a 5-mL Eppendorf tube for DNA extraction. Extraction of genomic DNA was performed using a genomic DNA extraction kit (RBC), following the manufacturer’s instructions. A spin column (QIAquick PCR purification kit; Qiagen) was utilized for DNA cleanup. The NanoDrop 2000 UV-visible (UV-vis) spectrometer was used for measuring the ratio of absorbance at 260/280 nm and 260/230 nm. The genomic DNA (gDNA) concentration was measured using a Qubit double-stranded DNA (dsDNA) high-sensitivity (HS) assay kit (Invitrogen) with a Qubit 2.0 fluorometer. The quantity and size distribution of the purified gDNA were calculated using Agilent 2200 TapeStation software (A.01.05) (6). A Covaris g-TUBE device was used to shear the genomic DNA according to the manufacturer’s specifications. Between 1 and 5 μg DNA was normalized for library construction using the PacBio SMRTbell 20-kb library preparation kit and sequenced on the PacBio Sequel platform (7). For bioinformatics analysis, default parameters were applied unless otherwise noted. In all, 558,605 PacBio raw reads (read length *N*_50_, 7,594 bp) were assembled *de novo* using Flye version 2.8.3 with the parameter “asm-coverage 100” (8). The resulting circular contig was polished using pbmm2 version 1.4.0 and GCpp version 2.0.2 until a highly accurate consensus for the final assembly was derived (https://github.com/PacificBiosciences/pbbioconda). Then, the genome was rotated using the fixstart method in Circlator version 1.5.5 (9). Finally, we verified that the genome of YSM-43^T^ was complete, with a circular form of 3,913,692 bp and a GC content of 29.38%. No plasmids were detected. The completeness of the genome assembly was assessed using BUSCO version 5.2.2 with the lineage flavobacteriales_odb10 data set (10). It showed that 732 of the 733 BUSCO groups were found to be complete, and 1 was fragmented. Subsequently, genomic annotation was conducted using the NCBI Prokaryotic Genome Annotation Pipeline (PGAP) version 6.1 (11). As a result, 3,537 coding sequences, 50 tRNAs, 9 rRNAs, and 3 noncoding RNAs (ncRNAs) out of a total of 3,599 genes were predicted. The complete circular genome map of *F. sediminilitoris* YSM-43^T^ was built using the Proksee server (https://proksee.ca/) (Fig. 1). To assign the Clusters of Orthologous Groups (COGs) functional categories in accordance with biological systems (12), eggNOG-mapper version 2.1.6 was used with the eggNOG v5 database (13, 14) (Table 1).

**TABLE 1:** General features of the complete genome sequence of Flavobacterium sediminilitoris YSM-43^T^

Genomic feature	Value	% of total	Description
Genome size (bp)	3,913,692		
DNA GC (%)	29.38		
No. of DNA scaffolds	1		
Total no. of genes	3,599		
No of CDSs	3,537		
No of rRNAs	9		
No of 16S rRNAs	3		
No of 23S rRNAs	3		
No of 5S rRNAs	3		
No of tRNAs	50		
No of ncRNAs	3		
No. of genes assigned to COGs			
Total	2,694		
M	275	8.94	Cell wall/membrane/envelope biogenesis
E	190	6.18	Amino acid transport and metabolism
J	176	5.72	Translation, ribosomal structure, and biogenesis
K	162	5.27	Transcription
H	141	4.58	Coenzyme transport and metabolism
L	141	4.58	Replication, recombination, and repair
O	138	4.49	Posttranslational modification, protein turnover, and chaperones
C	131	4.26	Energy production and conversion
T	120	3.9	Signal transduction mechanisms
P	107	3.48	Inorganic ion transport and metabolism
I	86	2.8	Lipid transport and metabolism
G	74	2.41	Carbohydrate transport and metabolism
F	68	2.21	Nucleotide transport and metabolism
V	55	1.79	Defense mechanisms
Q	45	1.46	Secondary metabolite biosynthesis, transport, and catabolism
U	43	1.4	Intracellular trafficking, secretion, and vesicular transport
D	27	0.88	Cell cycle control, cell division, and chromosome partitioning
N	17	0.55	Cell motility
W	3	0.1	Extracellular structures
A	1	0.03	RNA processing and modification
B	1	0.03	Chromatin structure and dynamics
Z	1	0.03	Cytoskeleton
S	692	22.5	Function unknown
UC^*a*^	382	12.42	Not in COGs

a UC, uncharacterized.

### Data availability.

The complete genome sequence of *F. sediminilitoris* YSM-43^T^ has been deposited at GenBank under the accession number CP090145. The raw data have been deposited in the SRA under the accession number SRR17867805.
